# Supplementary material for: Preventing acute asthmatic symptoms by targeting a neuronal mechanism involving carotid body lysophosphatidic acid receptors
Source: Nat Commun. 2018 Oct 2;9:4030. doi: 10.1038/s41467-018-06189-y (PMC6168495; doi:10.1038/s41467-018-06189-y)
Supplement: Supplementary file 1 — Supplementary Information [file 41467_2018_6189_MOESM1_ESM.pdf]

SUPPLEMENTARY INFORMATION

Preventing acute asthmatic symptoms by targeting a neuronal mechanism involving carotid body lysophosphatidic acid receptors

Jendzjowsky et al.

Online Supplement

Supplementary Table 1. Hemodynamic parameters before and after bradykinin challenge

Group	Time	HR (beats·min^{-1})^*	SBP (mmHg)	DBP (mmHg)	MAP (mmHg)	PaO2 (mmHg) ^*	PaCO2 (mmHg) ^*	pH^
Naïve	Pre	426±7	120±5	100±4	106±3	93±3	36±1	7.48±0.02
	Post	407±13	137±6$	117±8$	124±7$	82±5	37±1	7.43±0.02
OVA	Pre	378±12	116±6	95±10	102±8	72±5	46±1	7.37±0.02#
	Post	364±9	126±6	98±10	107±9	67±3	52±3%	7.32±0.03#
OVA + Carotid body denervation	Pre	390±10	114±5	100±4	105±3	78±4	42±3	7.36±0.02#
	Post	371±12	117±13	98±6	104±7	65±5	50±2	7.30±0.26#
OVA + Vagotomy	Pre	383±9#	117±5	108±3	111±3	81±5	44±3	7.39±0.02#
	Post	348±14#	113±7	104±5	107±5	65±5	49±4	7.33±0.04#
OVA + LPAr Blockade	Pre	365±9#	131±6	109±9	116±8	78±3	45±1	7.37±0.02#
	Post	357±8#	145±8$†	111±8	122±7	70±3	44±1	7.39±0.03#
OVA + TRPV1 Blockade	Pre	377±6#	117±7	98±6	104±5	73±3	41±1	7.37±0.01#
	Post	378±4#	121±4	105±5	110±4	63±3	44±1	7.35±0.01#
OVA + LPAr + TRPV1 Blockade	Pre	397±16	119±6	97±4	104±2	77±4	39±2	7.40±0.01#
	Post	394±18	127±6	108±5$	114±3$	75±7	38±2	7.36±0.02#

HR: $F_{6,41}$ (group)=3.876, p<0.004, ^. $F_{6,41}$ (time)=25.738, p<0.001, *.
SBP: $F_{6,79}$ (interaction)=2.399, p=0.049.
DBP: $F_{6,79}$ (interaction)=3.038, p=0.018.
MAP: $F_{6,79}$ (interaction)=3.175, p=0.014.
PaO_2: $F_{6,41}$ (group)=6.456, p<0.001, ^. $F_{6,41}$ (time)=45.593, p<0.001, *.
$PaCO_2$: $F_{6,41}$ (group)=5.615, p<0.001, ^. $F_{6,41}$ (time)=8.079, p=0.007, *.
pH: $F_{6,41}$ (group)=7.779, p<0.001, ^. $F_{6,41}$ (time)=19.103, p<0.001, *.
Holm-Šidák post-hoc test: pre- and post-values within group, p<0.05, $. Different than Vagotomy at same time point, p<0.05, †.
DOI: [10.6084/m9.figshare.6884810].

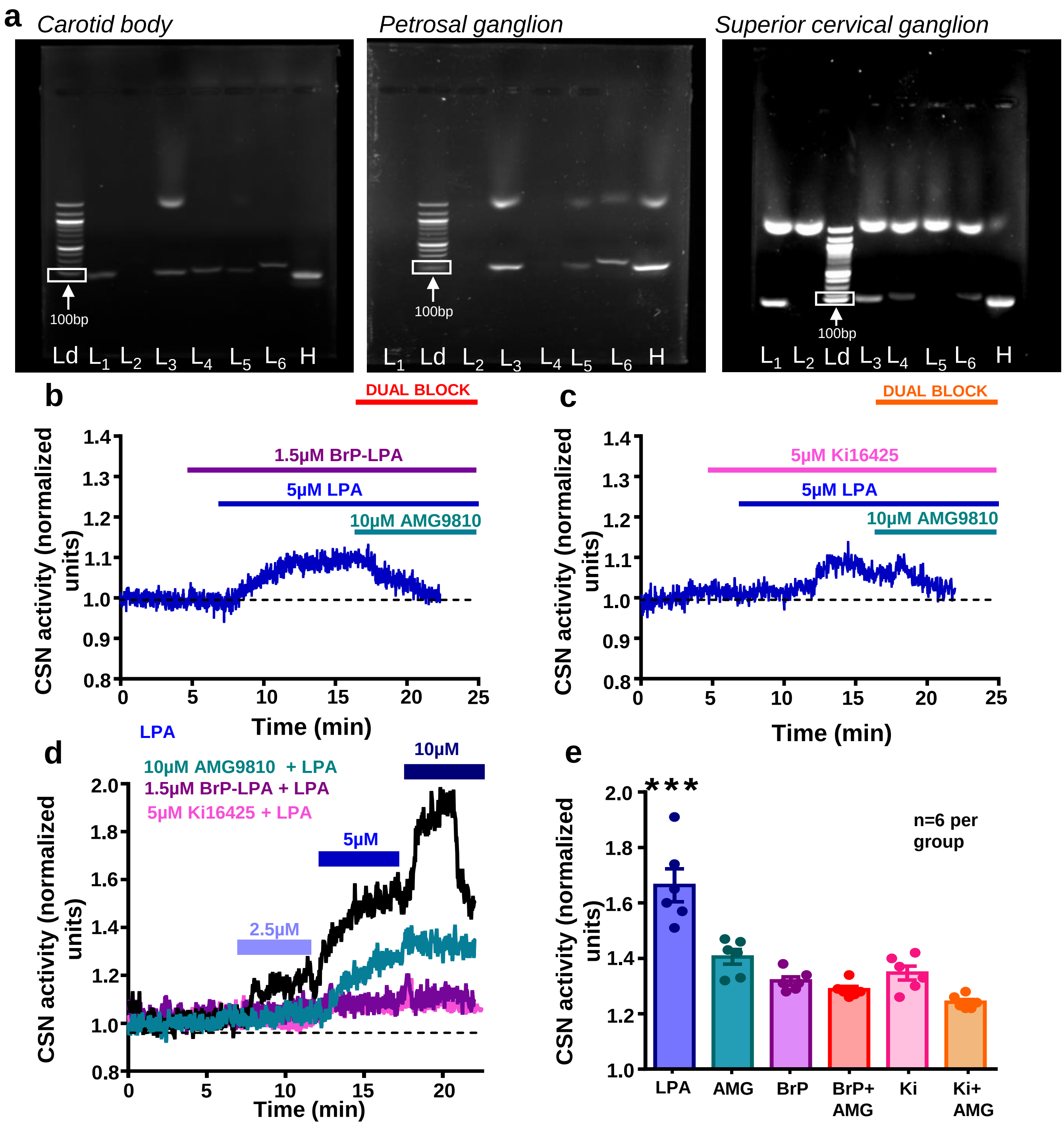

Supplementary Figure 1. **LPA-mediated carotid sinus nerve excitation.** a) Uncropped gels of RT-PCR. The expression of LPAr (L) 1, 3, 4, and 6 in the carotid body (left), LPAr 3 and 6 in the petrosal ganglia (centre), and LPAr 1, 3, 4, and 6 in the superior cervical ganglia (right) in reference to a housekeeping gene, hypoxyribosyltransferase (H; cDNA ladder lane, Ld, was used to identify tissues used for each gel) 100bp marker as indicated on respective gels.b, c) The application of BrP-LPA (1.5µM, mauve bar, a) or Ki16425 (5 µM, fuscia bar, b) diminishes the response to LPA (5µM, blue bar); the remaining response is almost abolished by subsequent application of AMG9810 (10µM, teal bar); Dual block portion is denoted by red (b) or orange (c) bar. d) Effects of TRPV1 and LPAR antagonists on CSN response to LPA. No blockade (black trace), TRPV1 blockade (AMG9810 10µM, teal trace) or LPAr blockade (BrP-LPA 1.5µM, mauve trace; Ki16425 5µM, fuscia trace). Data are from single experiments for each condition. e) Summary data of 5µM LPA (blue), with individual TRPV1 blockade (AMG 9810, 10µM; teal), individual LPAr blockade (BrP-LPA, 1.5µM; mauve; Ki16425, 5µM; fuscia), or combined LPAr and TRPV1 blockade (AMG9810+BrP red; AMG9810+Ki16425 orange), $F_{5, 35}$ (one-way ANOVA) =26.164, p<0.001. Holm- Šidák post hoc: AMG9810+LPA is significantly different from Ki16425+AMG9810+LPA, p=0.005; LPA is significantly different from all other groups, p<0.001, ***. All data are presented as mean±sem. DOI: [10.6084/m9.figshare.6882929].

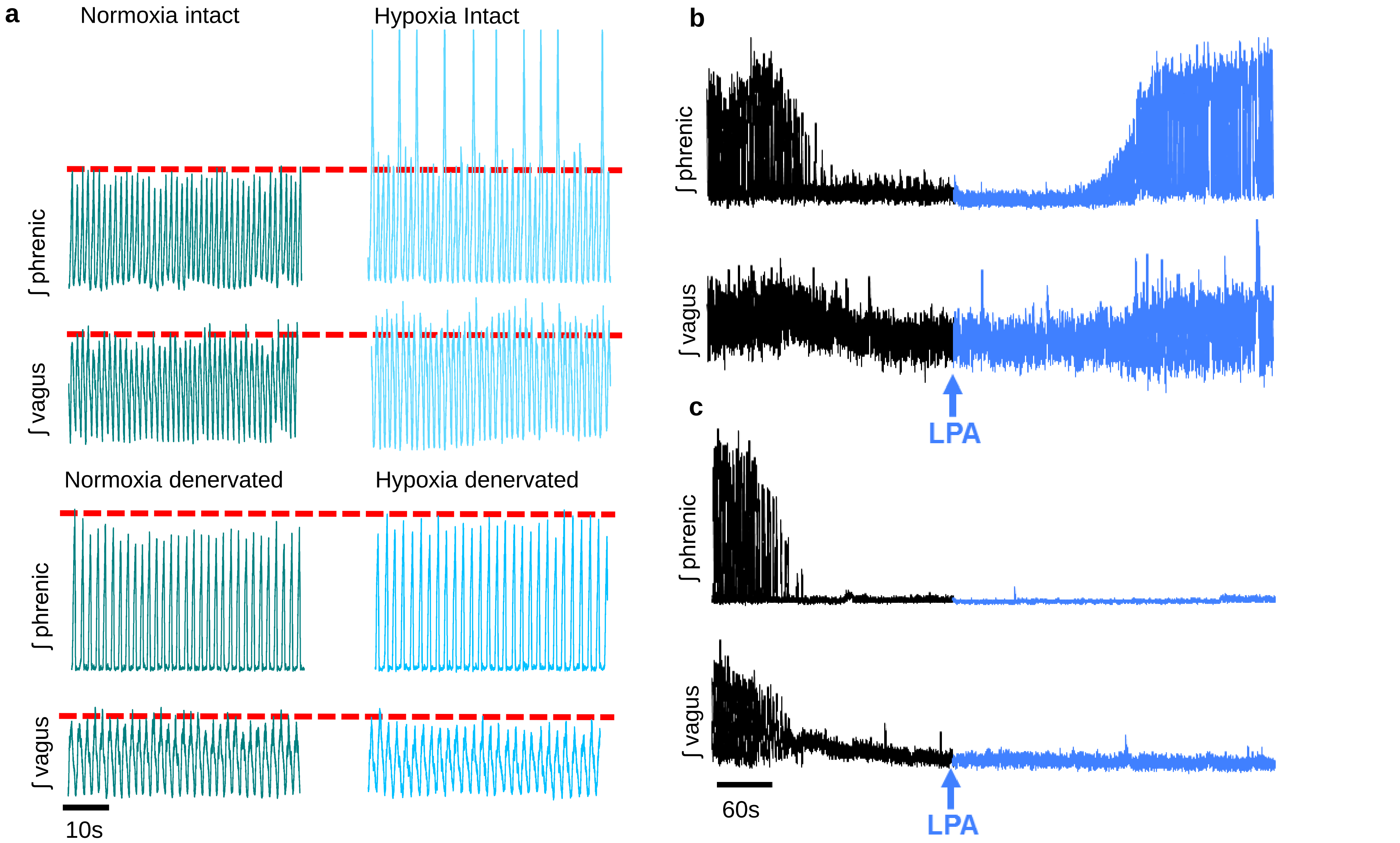

Supplementary Figure 2. **The dual-perfused preparation demonstrates carotid body stimulation causes increased efferent vagal activity.** This preparation allows independent artificial perfusion of carotid bodies and brainstem. a) Phrenic (top) and vagal (bottom) neural traces for a carotid body intact preparation under normoxic (dark teal) and hypoxic (light teal) conditions. Carotid sinus nerve resection does not affect normoxic (dark teal) but abolishes the hypoxic (light teal) response to both phrenic and vagal activity. b, c) The cessation of phrenic nerve firing and reduction of vagal activity in response to hypocapnia (solid; used to increase sensitivity of the preparation to carotid body activity) is reversed by carotid body injection of LPA (5µM, blue arrow/trace) in intact (b) but not denervated (c) preparations. DOI: [10.6084/m9.figshare.6882938].

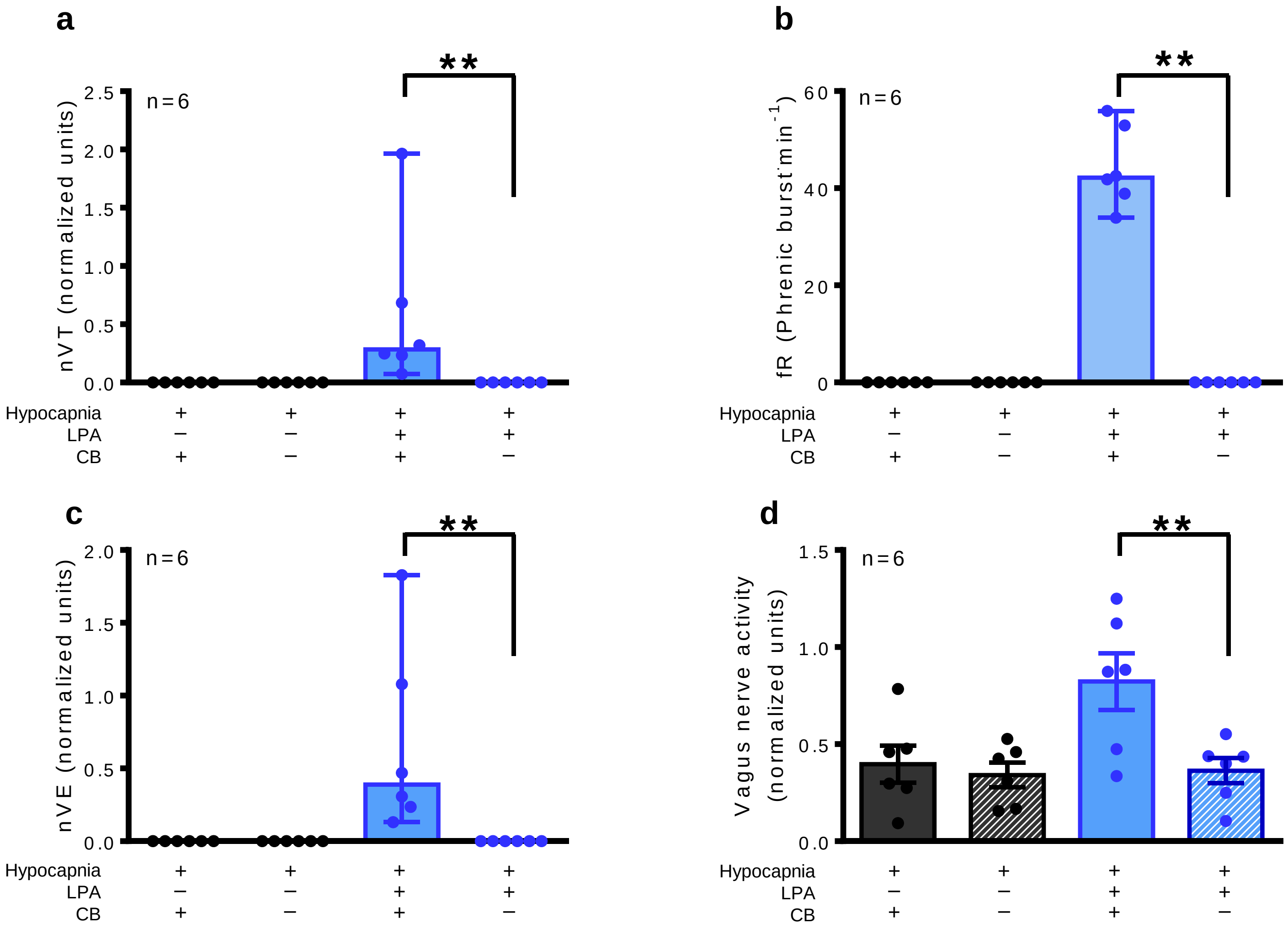

Supplementary Figure 3. **Summary data for dual-perfused preparation.** Data demonstrates carotid sinus nerve resection abrogates normal neuronal response to perfusing the carotid bodies with LPA (5µM; *blue*). Carotid sinus nerve intact, *filled bars*; resected, *hatched bars*. a) neural tidal volume (nVT, phrenic amplitude; ** Mann Whitney; U=0, p=0.002, **). b) neural frequency (fR, phrenic burst·min^{-1} Mann Whitney; U=0, p=0.002, **). c) neural minute ventilation (nVE, nVTx fR; ** Mann Whitney; U=0, p=0.002, **); and d) total vagal nerve activity (** Independent t-test: t_{10}=4.008, p=0.0025, **). All data except fR were normalized to the prior normoxic/normocapnic condition. Non-parametric data are presented as median±range, parametric data are presented as mean±sem. DOI: [10.6084/m9.figshare.6882938].

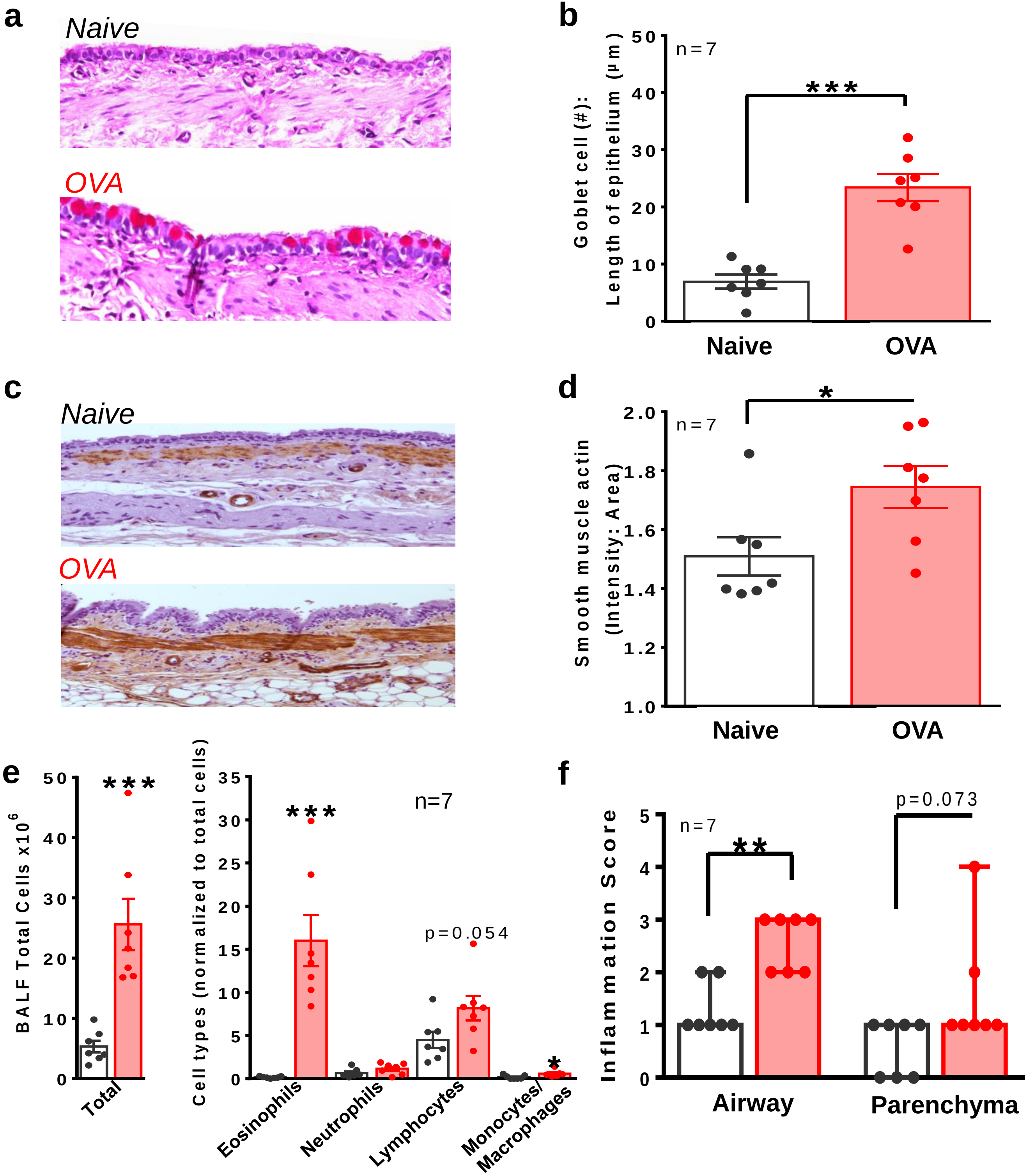

Supplementary Figure 4. **Increased indices of inflammation in ovalbumin-sensitized rats.** a) Periodic acid-Schiff's reagent stain demonstrating goblet cell metaplasia in ovalbumin sensitized (OVA, *bottom*) but not naïve (*top*) Brown Norway rats. b) Summary data of goblet cell metaplasia. Independent t-test: t_{12}=6.118, p<0.0001, ***. c) Smooth muscle actin immunohistochemistry demonstrating airway smooth muscle thickening in ovalbumin-sensitized (OVA, *bottom*) compared to naïve (*top*) Brown Norway rats. d) Summary data of smooth muscle actin analyzed as intensity of absorption/area of smooth muscle (see *Methods*). Independent t-test: t_{12}=2.430, p=0.032, *. e) BALF attained from naïve (open, n=7) and ovalbumin-sensitized (OVA, red, n=7) rats. Total leukocyte, eosinophil and macrophage count was significantly increased with ovalbumin sensitization with a trend for increased lymphocyte infiltration (data indexed to total cell count). Total cells: t_{12}=4.633, p<0.001, ***. Eosinophils: t_{12}=5.348, p<0.001, ***. Neutrophils: t_{12}=1.642, p=0.127. Lymphocytes: t_{12}=2.137, p=0.0539. Monocytes/macrophages: t_{12}=2.497, p=0.0280, *; all analyzed by independent t-test. f) Inflammatory score assigned to airway and parenchyma (see *Methods*) in naïve (*open*) and OVA (*red*) bars. Airway: Mann Whitney; U=3.0, p=0.004; Parenchyma Mann Whitney; U =10.0, p=0.073. Non-parametric data are presented as median±range, parametric data are presented as mean±sem. DOI: [10.6084/m9.figshare.6884810].

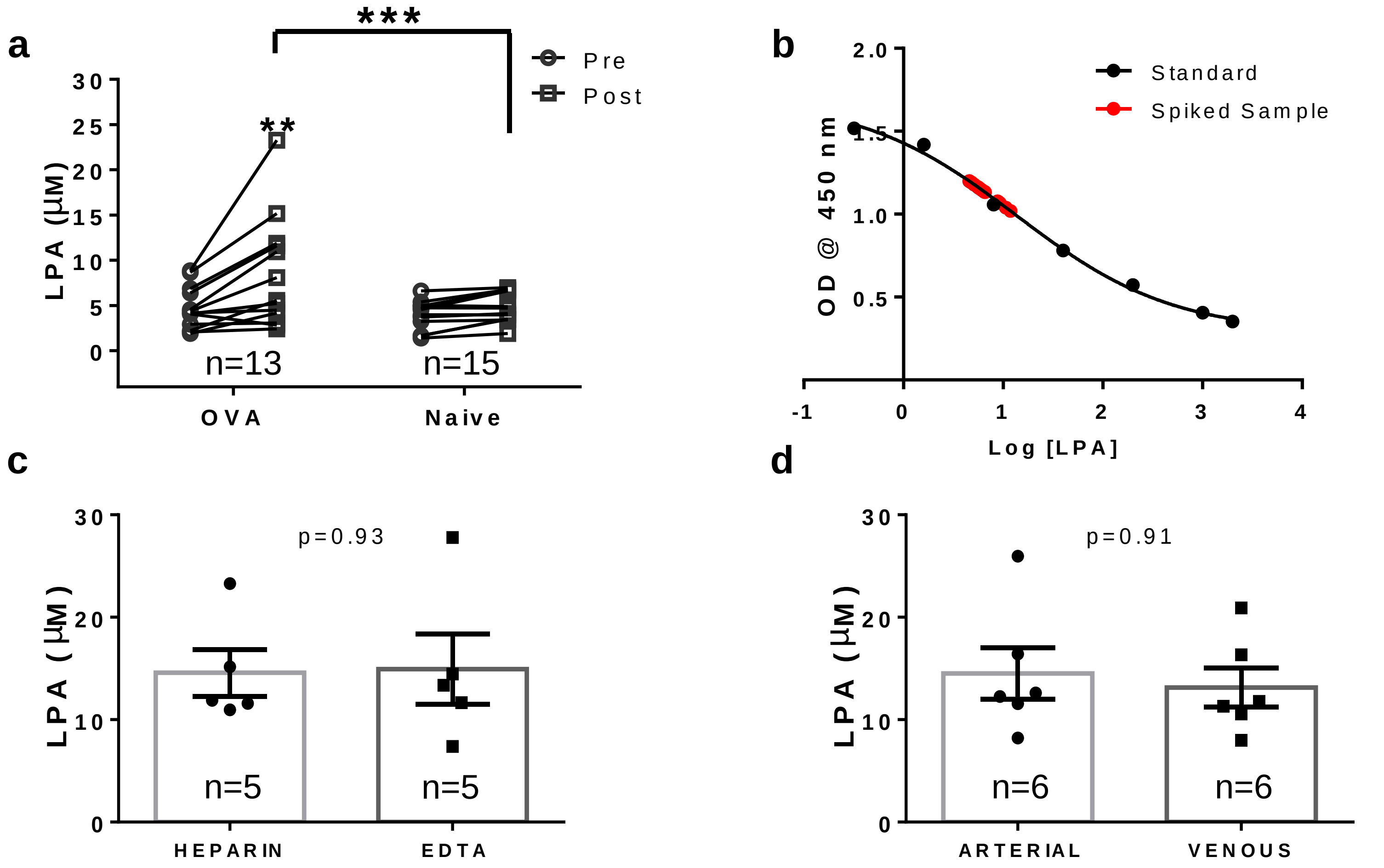

Supplementary Figure 5. **Ovalbumin-sensitized rats demonstrate increased LPA in plasma.** a) Bradykinin increases LPA in OVA animals only (see *Methods, OVA Cohort 1*; $F_{1,55}$ (two-way RM-ANOVA; time x group) =6.19, p=0.02). Holm- Šidák post hoc: bradykinin increases LPA in OVA rats (p<0.001, ***); bradykinin has no effect on LPA in naïve rats (p=0.21), LPA in naïve and OVA rats prior to bradykinin are not significantly different (p=0.58); but LPA in OVA rats is greater than naïve rats following bradykinin (p=0.01, **). b) The accuracy of ELISA analysis was validated with a plasma sample from an OVA-sensitized rat 'spiked' with different concentrations of known 18:1 LPA. The data fit on the log LPA curve generated with LPA standards provided by manufacturer. Pearson R=0.92, p<0.0001. c) Arterial plasma drawn from OVA-sensitized rats following bradykinin challenge demonstrated similar concentrations of LPA when treated with heparin (*circles*) or EDTA (squares). Independent t-test: t_8=0.09 p=0.93. d) Plasma LPA concentration did not differ in OVA-sensitized rats following bradykinin challenge between arterial (*circles*) or venous (*squares*) samples treated with heparin. Independent t-test: t_{10}=0.12 p=0.91. Data are presented as individual points and mean±sem. DOI: [10.6084/m9.figshare.6884687].

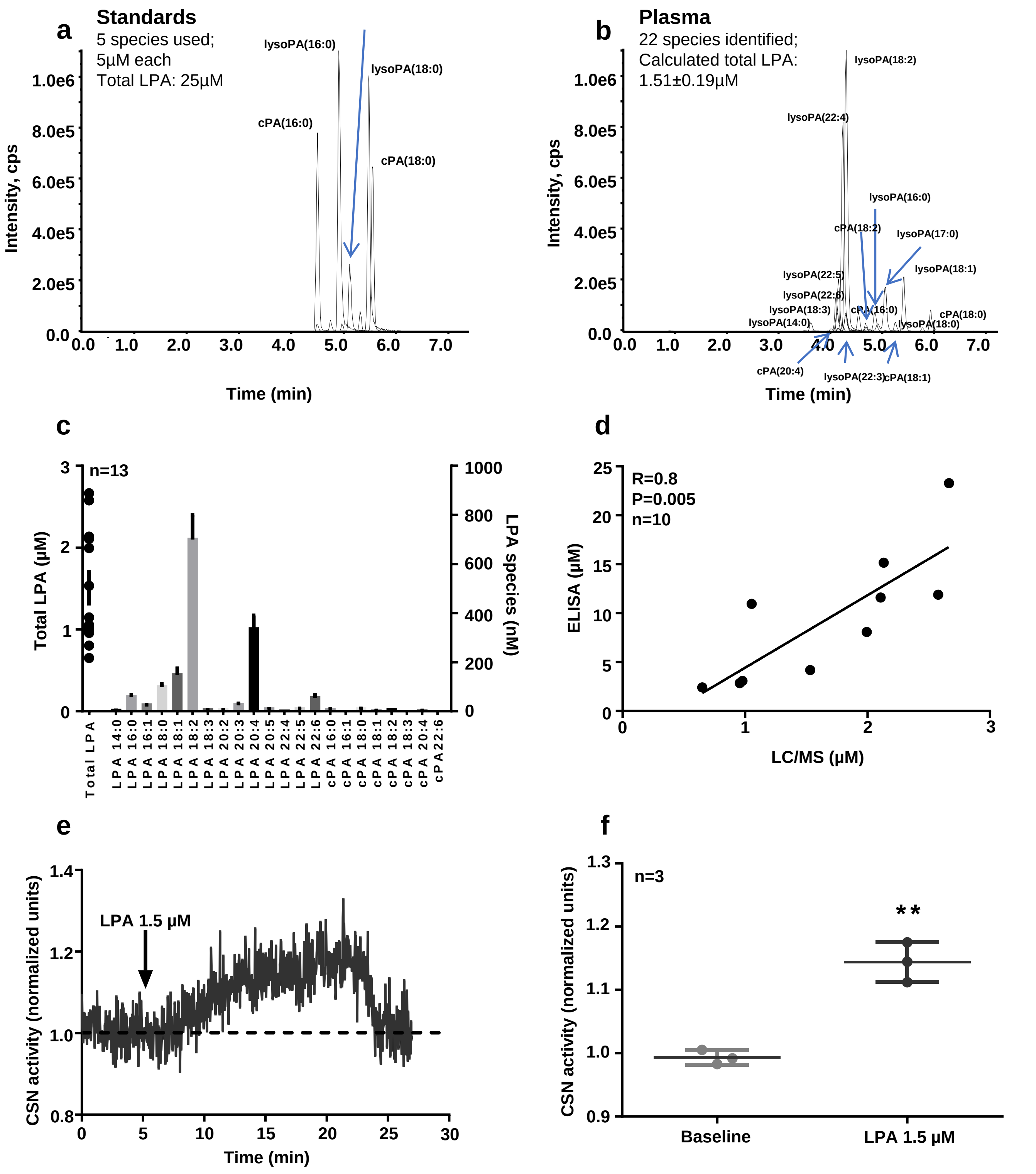

Supplementary Figure 6. **LC/MS measurement of arterial plasma from OVA rats following bradykinin.** a) LC/MS detection of LPA standards (LPA 16:0, 17:0, 18:0; cPA 16:0, 18:0; total LPA 25µM). b) LC/MS sample detection of all LPA species in plasma from a representative OVA challenged rat ~20 minutes following bradykinin. c) Summary of LC/MS detection of Total LPA (µM, left axis) and individual species (nM, right axis) in n=13 plasma samples from OVA rats following ~20 minutes bradykinin challenge (mean±sem). d) Correlation of total LPA concentration of the 10 samples measured by ELISA and LC/MS in OVA challenged rats ~20 minutes following bradykinin (Pearson correlation: R=0.8, p=0.005). e) Demonstration of a physiological effect of 1.5µM LPA on carotid sinus nerve activity in the en bloc carotid body preparation. f) Summary data of 1.5µM LPA versus baseline in the en bloc carotid body preparation showing a physiological effect of the concentration of LPA as measured by LC/MS (paired t-test: t_2=-9.794, p=0.01, mean±sem). DOI: [10.6084/m9.figshare.6884795].

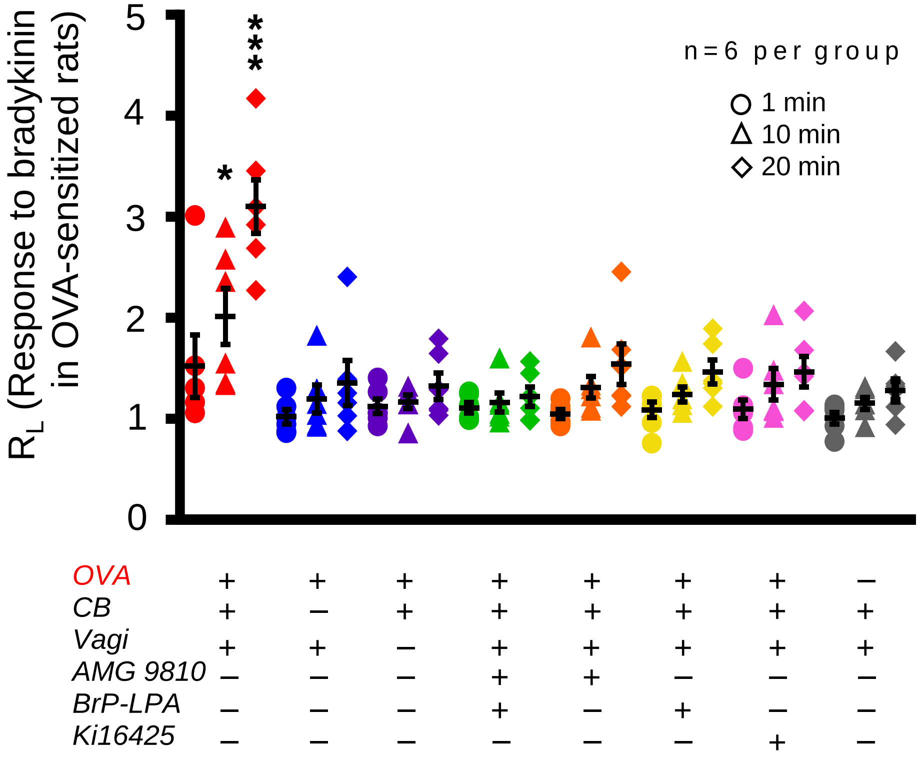

Supplementary Figure 7. **Bradykinin-induced bronchoconstriction in ovalbumin sensitized rats is dependent on the carotid body and LPA signalling.** OVA-sensitized and naïve rats were exposed to nebulized saline (baseline) and three consecutive nebulizations of 0.4mg bradykinin at 1 (circles), 10 (triangles) and 20 (diamonds) min while measuring RL. Bradykinin had group specific effects: See Methods, OVA Cohort 2; $F_{14,143}$ (two-way RM ANOVA: time x group) =4.035, $p<0.001$. Holm- Šidák post hoc: bradykinin caused a marked increase in RL in OVA-sensitized (red; $p<0.01$, **) but not naïve rats (white; $p>0.3$) rats; carotid body (CB) denervated (blue), vagi (VaG) denervated (purple), TRPV1 blockade (AMG9810, orange), LPAr blockade (BrP-LPA, yellow and Ki16425, fuscia) and dual TRPV1 and LPAr blockade (AMG9810 + BrP-LPA, green), abolished the effects of bradykinin compared to OVA (Holm- Šidák post-hoc: $p<0.05$, **; $p<0.001$, ***). Individual data are presented along with mean±sem. DOI: [10.6084/m9.figshare.6884891].

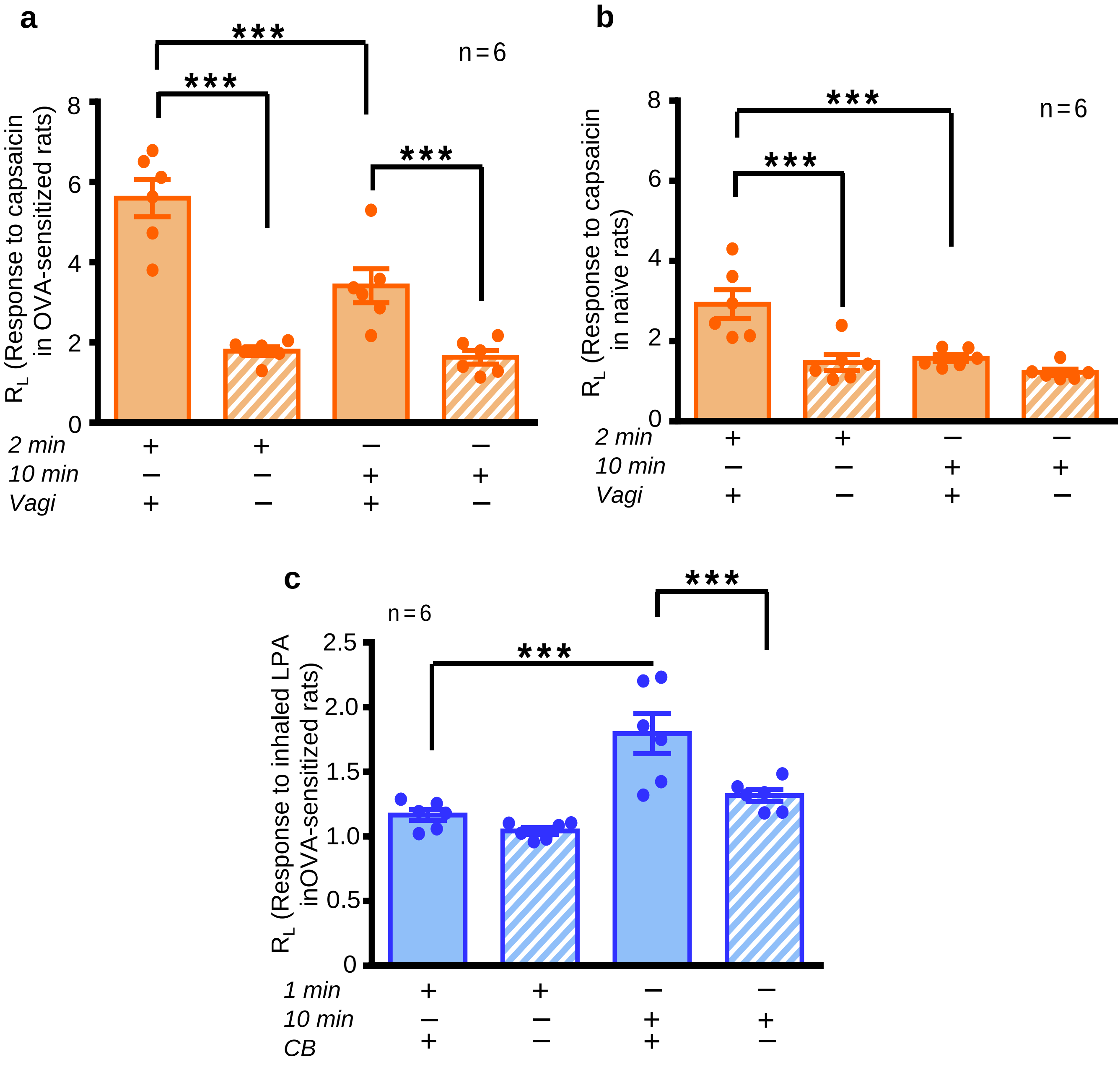

Supplementary Figure 8. **Vagal-vagal and local reflexes in OVA and naïve rats.** a, b). Bronchoconstriction in response to capsaicin does not involve the carotid body (see *Methods, OVA Cohort 4*). R_L was measured 2 and 10 min after single capsaicin nebulization in OVA (*a*) and naïve (*b*) rats who had intact (*solid*) or resected vagi (*hatched*). All rats had denervated carotid bodies yet all, including naïve animals, exhibited bronchoconstriction in response to capsaicin by 2 min. OVA-sensitized rats (a) $F_{1,23}$ (two-way RM-ANOVA time x group) =12.638, p=0.005. Naïve rats (b) $F_{1,23}$ (two-way RM-ANOVA time x group) =10.323, p=0.009. Holm- Šidák post hoc: difference from vagotomy, and 2 & 10min, p<0.001, ***. c) Inhaled LPA (18:1, 5µM) causes delayed (after 30min) bronchoconstriction in OVA-sensitized rats which is dependent on the carotid body (see *Methods*, *OVA Cohort 5*). $F_{1,10}$ (two-way RM-ANOVA group) =13.363, p=0.004. $F_{1,1}$ (two-way RM-ANOVA time) =26.447, p<0.001. Holm- Šidák post hoc: 1 and 30 min different in carotid body intact group, p<0.001, ***. Carotid body intact different from carotid body denervated group at 30 min, p<0.001, ***. All data are presented as mean±sem. DOI: [10.6084/m9.figshare.6884891].

Supplementary Table 2. Primer sequences

Gene	Sense (5’-3’)	Anti-sense (5’-3’)	Size (bp)
LPAr1	GAC ACC ATG ATG AGC CTT CTG A	CCC GGA GTC CAG CAG AGA	71
LPAr2	CCA GCC TGC TTG TCT TCC TA	GTG TCC AGC ACA CCA CAA AT	180
LPAr3	CAC ACG AGT TTC TCC ATC AG	GGT CCA GCA CAC CAC GAA	93
LPAr4	TCT GGA TCC TAG TCC TCA GTG G	CAG ACT TCC TGT GCC AGA CG	107
LPAr5	CAG ACT TCC TGT GCC AGA CG	TAG CGG TCC ACG TTG ATG AG	91
LPAr6	TCT GGC AAT TGT CTA CCC ATT	TCA AAG CAG GCT TCT GAG G	165
HPRT	CCT TGA CTA TAA TGA GCA CTT C	GCC ACA TCA ACA GGA CTC TTG TAG	126
IL4	GTG ATG TAC CTC CGT GCT TG	TTG TGA GCG TGG ACT CAT TC	123
CCL11	CAC GGC CAC TTC CTT CAC	CAG CGT GCA TCT GTT GTT G	142
β-actin	GGA TGT CAA CGT CAC ACT TCA	CAG GTC ATC ACT ATC GGC AA	133
